# Valorization of Lipids from *Gracilaria* sp. through Lipidomics and Decoding of Antiproliferative and Anti-Inflammatory Activity

**DOI:** 10.3390/md15030062

**Published:** 2017-03-02

**Authors:** Elisabete da Costa, Tânia Melo, Ana S. P. Moreira, Carina Bernardo, Luisa Helguero, Isabel Ferreira, Maria Teresa Cruz, Andreia M. Rego, Pedro Domingues, Ricardo Calado, Maria H. Abreu, Maria Rosário Domingues

**Affiliations:** 1 Centro de Espectrometria de Massa, Departamento de Química & QOPNA, Universidade de Aveiro, Campus Universitário de Santiago, 3810-193 Aveiro, Portugal; elisabetecosta@ua.pt (E.d.C.); taniamelo@ua.pt (T.M.); ana.moreira@ua.pt (A.S.P.M.); p.domingues@ua.pt (P.D.); 2 Instituto de Biomedicina (IBIMED), Departamento de Ciências Médicas, Universidade de Aveiro, 3810-193 Aveiro, Portugal; carinabernardo@ua.pt (C.B.); luisa.helguero@ua.pt (L.H.); 3 Centro de Neurociências e Biologia Celular (CNC), Universidade de Coimbra, 3004-517 Coimbra & Faculdade de Farmácia, Universidade de Coimbra, 3000-548 Coimbra, Portugal; isabelcvf@gmail.com (I.F.); trosete@ff.uc.pt (M.T.C.); 4 ALGAplus-Produção e Comercialização de Algas e seus Derivados, Lda., 3830-196 Ílhavo, Portugal; amrego@algaplus.pt (A.M.R.); htabreu@algaplus.pt (M.H.A.); 5 Departamento de Biologia & CESAM, Universidade de Aveiro, Campus Universitário de Santiago, 3810-193 Aveiro, Portugal; rjcalado@ua.pt

**Keywords:** glycolipids, phospholipids, betaine lipids, seaweeds, bioactivity, mass spectrometry, hydrophilic interaction liquid chromatography–electrospray ionization–mass spectrometry HILIC–ESI–MS

## Abstract

The lipidome of the red seaweed *Gracilaria* sp., cultivated on land-based integrated multitrophic aquaculture (IMTA) system, was assessed for the first time using hydrophilic interaction liquid chromatography-mass spectrometry and tandem mass spectrometry (HILIC–MS and MS/MS). One hundred and forty-seven molecular species were identified in the lipidome of the *Gracilaria* genus and distributed between the glycolipids classes monogalactosyl diacylglyceride (MGDG), digalactosyl diacylglyceride (DGDG), sulfoquinovosyl monoacylglyceride (SQMG), sulfoquinovosyl diacylglyceride (SQDG), the phospholipids phosphatidylcholine (PC), lyso-PC, phosphatidylglycerol (PG), lyso-PG, phosphatidylinositol (PI), phosphatidylethanolamine (PE), phosphatic acid (PA), inositolphosphoceramide (IPC), and betaine lipids monoacylglyceryl- and diacylglyceryl-*N,N,N*-trimethyl homoserine (MGTS and DGTS). Antiproliferative and anti-inflammatory effects promoted by lipid extract of *Gracilaria* sp. were evaluated by monitoring cell viability in human cancer lines and by using murine macrophages, respectively. The lipid extract decreased cell viability of human T-47D breast cancer cells and of 5637 human bladder cancer cells (estimated half-maximal inhibitory concentration (IC_50_) of 12.2 μg/mL and 12.9 μg/mL, respectively) and inhibited the production of nitric oxide (NO) evoked by the Toll-like receptor 4 agonist lipopolysaccharide (LPS) on the macrophage cell line RAW 264.7 (35% inhibition at a concentration of 100 μg/mL). These findings contribute to increase the ranking in the value-chain of *Gracilaria* sp. biomass cultivated under controlled conditions on IMTA systems.

## 1. Introduction

Red seaweeds within the genus *Gracilaria* are one of the world’s most cultivated and valuable marine macrophytes. This group of seaweeds is well adapted to cultivation on land-based integrated multitrophic aquaculture (IMTA) systems, allowing its sustainable production under controlled and replicable conditions that provide a secure supply of high-grade seaweed biomass for demanding markets (e.g., food, pharmaceuticals) [1,2,3]. *Gracilaria* sp. is a source of multiple products, among which lipids, namely polyunsaturated fatty acids (PUFAs) such as arachidonic (20:4(*n*-6), AA) and eicosapentaenoic (20:5(*n*-6), EPA) acids, are emerging as valuable components [4]. Fatty acids (FAs) are mainly esterified to polar lipids such as glycolipids, phospholipids, and betaine lipids. Polar lipids are nowadays recognized as an important reservoir of fatty acids with nutritional value, e.g., *n*-3 FAs [5,6], and they are also considered high-value novel lipids with beneficial health effects such as antitumoral [7,8], antiviral [8,9], antifungal [10], antibacterial [11], and anti-inflammatory [11,12], with potential applications in the nutraceutical and pharmaceutical industries [7,8,11]. However, in spite of their recognized potential, they are still scarcely studied [12,13,14,15]. Some studies reported that polar lipids isolated from seaweeds can promote growth-inhibiting effects on human hepatocellular carcinoma cell lines (HepG2) [16] and thus can act as inhibitors of DNA polymerases with capability to inhibit tumor cell proliferation [17]. Moreover, they have been associated with anti-inflammatory properties through the inhibition of pro-inflammatory cytokines interleukin IL-6 and IL-8 production [15] and/or by the inhibition of nitric oxide (NO) production [18,19,20,21]. Lipid-based agents are therefore emerging molecules in therapeutics aimed to regulate inflammatory pathways or even impair downstream tumorigenic processes [22,23,24].

To fully explore the bioactive properties of seaweed lipids and thus contribute to seaweed valorization, it is fundamental to characterize their structure and understand how it modulates bioactivity [13,14]. Nowadays, the detailed structural characterization of lipids can be accomplished by using mass spectrometry (MS) coupled with liquid chromatography (LC). This lipidomic approach has the advantage of providing a detailed analysis of the lipid profile and affording the identification and quantification of more than 200 lipid molecular species in one single LC–MS run [25,26]. This detailed information on the specificity of molecular species and corresponding classes of polar lipids cannot be achieved using traditional approaches, typically based on the previous separation of polar lipid classes by thin-layer chromatography (TLC) and silica gel on column chromatography, followed by off-line gas chromatography-mass spectrometry (GC–MS) analysis of FAs [27,28,29,30,31,32,33]. MS-based technologies allowed researchers to explore the full lipidomic signature of distinct matrices [34,35,36]. To date, they allowed for the identification of the full lipidome signature of cultivated seaweeds *Ulva lactuca* Linnaeus, 1753 [37], *Chondrus crispus* Stackhouse, 1797 [26], and *Codium tomentosum* Stackhouse, 1797 [25]. These novel approaches based on specific identification and quantification at the molecular level using high-throughput analysis are promising tools for bioprospection [3,38,39].

The main goal of the present study was to identify and characterize the polar lipid profile of *Gracilaria* sp. cultivated under controlled conditions on a land-based integrated multitrophic aquaculture (IMTA) system, using hydrophilic interaction liquid chromatography-electrospray ionization-mass spectrometry (HILIC–ESI–MS). The lipid extract of this red seaweed was also screened for its growth inhibitory effects in human breast and bladder cancer cell lines, as well as anti-inflammatory effects by inhibiting the production of NO.

## 2. Results and Discussion

The lipid extract of *Gracilaria* sp. obtained by chloroform:methanol extraction accounted for about 3000 ± 600 mg/kg dry mass (relative standard deviation (RSD) < 20%). The lipid extract was mainly composed of glycolipids (1980 ± 148 mg/kg of biomass) and phospholipids (165 ± 53 mg/kg of biomass), and the remaining lipid extract corresponded to betaine lipids and others (Table 1).

### 2.1. Polar Lipidome

**Figure 1:**
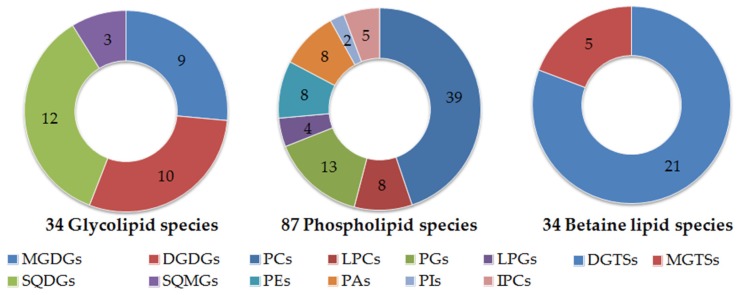
Number of molecular species identified by HILIC–ESI–MS, distributed by the classes of glycolipids: monogalactosyl diacylglyceride (MGDG), digalactosyl diacylglyceride (DGDG), sulfoquinovosyl monoacylglyceride (SQMG), sulfoquinovosyl diacylglyceride (SQDG), phospholipids: phosphatidylcholine (PC) and lyso-PC (LPC), phosphatidylglycerol (PG) and lyso-PG (LPG), phosphatidylinositol (PI), phosphatic acid (PA), phosphatidylethanolamine (PE), inositolphosphoceramide (IPC), and betaine lipids: monoacylglyceryl- and diacylglyceryl-*N*,*N*,*N*-trimethyl homoserine (MGTS and DGTS).

The profile of *Gracilaria* polar lipidome was determined by HILIC–ESI–MS and allowed for the identification of molecular species of glycolipids, phospholipids, and betaine lipids. Overall, the lipidome of *Gracilaria* sp. comprised 147 molecular species (Figure 1). 

The glycolipids of the classes monogalactosyl diacylglyceride (MGDG) and digalactosyl diacylglyceride (DGDG) were identified in the LC–MS spectra in positive mode as [M + NH_4_]^+^ ions [25]. Detailed structure of MGDG and DGDG molecular species was accomplished by LC–MS/MS analysis of [M + NH_4_]^+^ ions and analysis of ESI–MS/MS of the [M + Na]^+^ ions after solid phase extraction (SPE) fractionation of lipid extract (fraction 3 rich in glycolipids). Overall, 34 molecular species were identified, as described in Table 2. Galactolipids contained nine MGDG molecular species and 10 DGDG molecular species (Table 2, Figure S1a,b). The most abundant MGDG molecular species were found at *m/z* 774.3 and 796.3 [M + NH_4_]^+^, corresponding to MGDG (18:1/16:0) and to MGDG (20:4/16:0), with a minor contribution from MGDG (18:2/18:2), respectively. Other MGDG molecular species identified contained in their composition 14-, 16-, and 18-carbon saturated fatty acids (SFAs) and monounsaturated fatty acids (MUFAs) and 18:2, 20:4, and 20:5 polyunsaturated fatty acyl (PUFAs) moieties (Table 2). Regarding DGDGs, the most abundant molecular species were identified as [M + NH_4_]^+^ ions at *m/z* 936.3, corresponding to DGDG (18:1/16:0), followed by DGDG (20:4/16:0) with minor contribution of DGDG (18:2/18:2) at *m/z* 958.2. Moreover, other DGDG molecular species were identified containing 14-, 16-, 18-, and 20-carbon fatty acids (FAs) such as 20:4 and 20:5 PUFAs.

Concerning sulfolipids, 12 sulfoquinovosyl diacylglycerides (SQDGs) and three sulfoquinovosyl monoacylglycerides (SQMGs) were identified as negative [M − H]^−^ ions. The most abundant species were attributed to SQDG (14:0/16:0), SQDG (16:0/16:0) and SQDG (16:0/20:4), observed as [M − H]^−^ ions at *m/z* 765.5, 793.5, and 841.6, respectively. The fatty acyl signature of SQDGs included 14-, 16-, 18-carbon SFAs and MUFAs and 18- and 20-carbon PUFAs (Table 2, Figure S1c). Three SQMGs were identified as SQMG (14:0), SQMG (16:0), and SQMG (16:1). SQMGs were never before reported in the lipidome of seaweeds from the genus *Gracilaria*. Glycolipids have already been identified for members of the Rhodophyta (red seaweeds), namely in the genus *Gracilaria* [21,30,40,41,42]. However, the majority of published works only identified a few species of glycolipids, either by using offline TLC–MS [30,40,42,43] or selected solvent extraction and MS analysis [15,20,29]. More recently, a detailed profile of *Chondrus crispus* was reported using LC–MS and MS/MS [26].

*Gracilaria* sp. lipidome included 87 molecular species of phospholipids (PLs) within eight classes, namely phosphatidylglycerol (PG) and lyso-PG (LPG), phosphatidylcholine (PC) and lyso-PC (LPC), phosphatidylethanolamine (PE), phosphatidylinositol (PI), inositolphosphoceramide (IPC) and phosphatidic acid (PA). PC is a main component of extraplastidial membranes, while PG is found in chloroplastic membranes [5]. 

The PL classes PG, lyso-PG, PA, PI, and IPC were identified as negative [M − H]^−^ ions, while PC and lyso-PC were identified as negative [M + CH_3_COO]^−^ ions. PC, LPC and PE were also identified as positive [M + H]^+^ ions. The identity of all molecular species identified (Table 3) was confirmed by LC–MS/MS, as described in the literature [25,26]. About 39 PCs were identified by LC–MS (Figure S2a). The most abundant ions were observed at *m/z* 760.6 and at *m/z* 782.6, respectively attributed to PC (16:0/18:1) and to PC (16:0/20:4) with a minor contribution of PC (18:2/18:2). Other PC molecular species were identified and contained 14- to 22-carbon fatty acids. Lyso-PC consisted of eight molecular species (Table 3, Figure S2b) and the most abundant was LPC (20:4), observed at *m/z* 544.4. All molecular species identified are described in Table 3. Thirteen PGs and four lyso-PGs species were identified by LC–MS as [M – H]^−^ ions (Table 3, Figure S2c,d). The most abundant ion was observed at *m/z* 769.4, mainly corresponding to PG (16:0/20:4), with a minor contribution from PG (18:2/18:2). The prominent lyso-PG at *m/z* 483.3 was LPG (16:0). PI species were observed as [M − H]^−^ ions at *m/z* 833.5 and 835.5 and attributed to PI (16:1/18:1) and PI (16:0/18:1), respectively. Eight PAs were identified (Table 3, Figure S2e), with the most abundant species identified as PA (20:4/20:4) at *m/z* 743.3, while the other PA molecular species were esterified to 16:0, 18:1, 18:2, 18:3, 20:3, 20:4, and 20:5 FAs. PEs contained eight molecular species, identified as [M + H]^+^ (Table 3, Figure S2f). The most abundant ion was observed at *m/z* 716.4 and identified as PE (16:1/16:1) and PE (16:0/18:2). The phospholipids from *Gracilaria* sp. hold PCs and LPCs, PGs, LPGs, PIs, PEs, and PAs, already reported for the lipidome of other Rhodophyta [20,26,31,32]. Fatty acids esterified in the PLs included saturated and unsaturated 16-, 18-, and 20-carbon FAs, and PCs and PAs were the only PLs classes that included 20:3(*n*-6) FA. The 20:3(*n*-6) FA is usually a minor component of the whole pool of FAs in red seaweeds [31,32] but is an important intermediate compound in the biosynthesis of 20:4(*n*-6) FA. 

Five molecular species were assigned as IPCs and the most abundant ones were IPC (*t*18:0/17:0), observed at *m/z* 810.5, and IPC (*t*18:1/24:1), observed at *m/z* 920.6 (Figure S3a). LC–MS/MS spectrum of the [M − H]^−^ ions of IPCs, as exemplified for IPC at *m/z* 920.6 in Figure S3b, showed the typical fragmentation pathways of IPCs such as the losses of 162 Da and 180 Da, due to the fragmentation pathways that lead to the elimination of inositol, the product ion at *m/z* 538.3 resulting from the loss of fatty acyl chains, and the product ion at *m/z* 259.0 that corresponded to an inositol monophosphate anion. IPCs were identified in lipid extract of *Gracilaria* sp. and are considered an important biomarker of Rhodophyta taxonomy, in accordance with what was already reported for *Chondrus crispus* using a lipidomic approach [26]. IPCs are required to maintain membrane properties such as viscosity and electrical charge and participate in the control of enzymatic activity or act as membrane anchors for some proteins [44].

Twenty-one DGTS and five MGTS molecular species were identified by LC–MS and MS/MS as [M + H]^+^ ions (Table 4, Figure S4a,b, respectively). The most abundant DGTS species were found at *m/z* 710.7, corresponding to DGTS (16:0/16:1), with a minor contribution from DGTS (14:0/18:1) species, followed by DGTS (18:1/18:1) observed at *m/z* 764.8. Overall, DGTSs combine distinctive molecular species bearing different combinations of FAs, ranging between 14- and 20-carbon FAs, as reported on Table 4. MGTSs comprised MGTS (14:0), MGTS (16:1), MGTS (16:0), MGTS (18:2), and MGTS (18:1) species, identified at *m/z* 446.5, 472.5, 474.5, 498.6, and 500.6, respectively.

Betaine lipids are components of extraplastidial membranes [45]. They are naturally occurring lipids not found in higher plants, but are widely distributed in algae [45,46]. Betaines are a class of acyl glycerolipids that have a quaternary amine alcohol ether-linked to a diacylglycerol moiety, lacking in phosphorous. Interestingly, DGTSs are described herein for the first time in the lipidome of genus *Gracilaria* and MGTS species were not reported before in the lipidome of red seaweeds. This may be due to the lack of sensitivity of most reported analytical tools based on TLC and GC–MS approaches, since the co-elution of betaines and PC by TLC approaches could have prevented their discrimination [47]. Only recently, through the use of MS-based tools, betaine lipids were identified in the lipidome of red seaweed *Chondrus crispus* [26] and green seaweed *Codium tomentosum* [25].

### 2.2. Fatty Acid Profile

**Figure 2:**
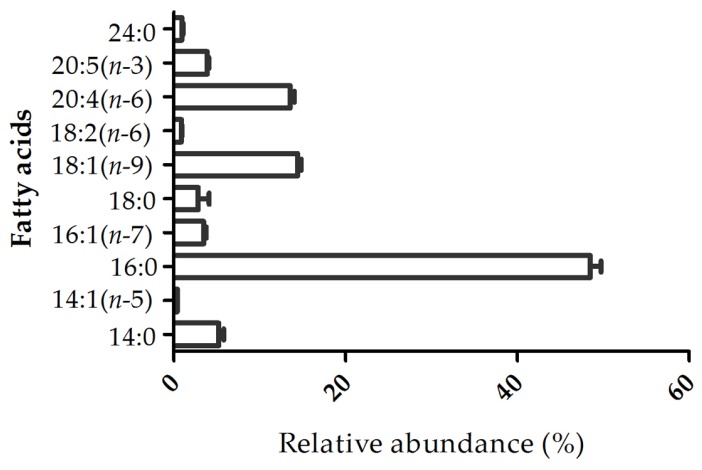
Fatty acid profile of lipids from *Gracilaria* sp. determined by GC–MS analysis of fatty acid methyl esters (FAMEs). Mean ± SD (%) of triplicate, traces < 0.1% not shown.

The fatty acid profile of the lipid extract was characterized by GC–MS analysis of fatty acid methyl esters (FAMEs). The profile of fatty acids included 14:0, 16:0, 18:0, 18:1(*n*-9), 18:2(*n*-6), 20:4(*n*-6), and 20:5(*n*-3), among which 16:0 (48.5% ± 1.1%), 18:1(*n*-9) (14.4% ± 0.38%), and 20:4(*n-*6) (13.6% ± 0.46%) were the most abundant (Figure 2). Overall, SFAs accounted for 57.5% of the total FAs identified, followed by MUFAs (18.3%) and PUFAs (18.4%). The fatty acids identified by GC–MS were also reported to be esterified to polar lipids of *Gracilaria* sp. by LC–MS and MS/MS analysis.

The *n*-6/*n*-3 ratio determined for our *Gracilaria* sp. sample was 3.6. The World Health Organization (WHO) recommends an optimal balance intake of *n*-6 PUFAs and *n*-3 PUFAs to prevent chronic diseases and that this balance should be maintained with an adequate daily dosage of *n*-6 PUFAs (5%–8% of daily energy intake) and *n*-3 PUFAs (1%–2% of daily energy intake) [48]. With this recommendation in mind, it is possible to estimate that a suitable *n*-6/*n*-3 ratio is less than 5. Also, some authors reported that a ratio of *n*-6/*n*-3 less than 4 is adequate in the prevention of several diseases such as cardiovascular [49], autoimmune [50], and inflammatory diseases [50,51], and cancer [49,50]. These findings support the use of *Gracilaria* sp. for human consumption.

### 2.3. Bioactivity of Lipid Extract of Gracilaria sp.

The bioactivity of *Gracilaria* sp. lipid extract was assessed, specifically its antiproliferative effect in two human cancer cell lines (breast cancer—T-47D and bladder cancer—5637) and its anti-inflammatory effect in a mouse leukemic monocyte macrophage cell line (RAW 264.7) stimulated with LPS. 

#### 2.3.1. Activity of Lipid Extract on Human Cancer Cell Viability

**Figure 3:**
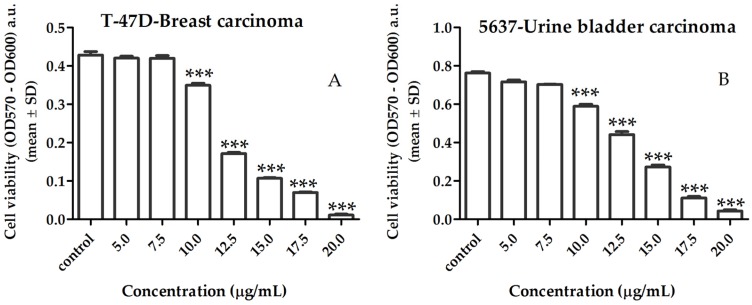
Effect of lipid extracts of *Gracilaria* sp. on T-47D breast (**A**) and 5637 bladder (**B**) cancer cell lines, after 96 h incubation. Results are shown as mean ± SD of three independent determinations (*** *p* < 0.001, compared to control). OD: optical density; a.u.: arbitrary units.

The growth inhibitory effect induced by the lipid extract on cancer cells is shown in Figure 3. A lipid extract of *Gracilaria* sp. reduced cell viability in both cell lines in a dose-dependent manner at concentration range of 10 to 20 μg/mL (*p* < 0.001), with a calculated half-maximal inhibitory concentration (IC_50_) of 12.2 μg/mL and 12.9 μg/mL for T-47D (Figure 3A) and 5637 (Figure 3B) cancer cells, respectively.

The anti-tumor effects of polar lipids were previously reported as affecting angiogenesis and solid tumor growth via inhibition of replicative DNA polymerase activities [22,52]. Extracts rich in glycolipids isolated from distinct seaweeds inhibited the growth of a human hepatocellular carcinoma cell line (HepG2) (IC_50_ of 126 μg/mL) [16] and were found to induce apoptosis of human colon carcinoma Caco-2 cells when associated with sodium butyrate [53]. Otherwise, SQDG isolated from *Gigartina tenella* Harvey, 1860, accepted as *Chondracanthus tenellus* (Harvey) Hommersand, 1993, inhibited DNA polymerase α, DNA polymerase β, and HIV-reverse transcriptase type 1 or downregulated *Tie2* gene expression in tumors [17,54]. It has been hypothesized that the biological properties of glycolipids such as SQDG are closely related to the sugar moiety and the presence of PUFA chains.

#### 2.3.2. Activity of the Lipid Extract on Nitric Oxide Production

**Figure 4:**
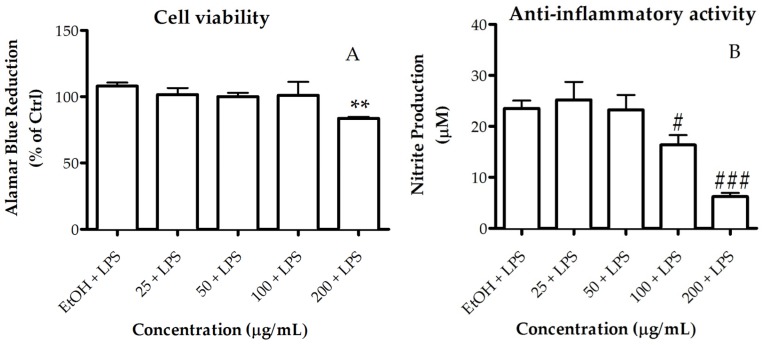
Cell viability and anti-inflammatory activity of *Gracilaria* sp. lipid extract. (**A**) Assessment of metabolically active cells was performed using a resazurin bioassay. Results are expressed as a percentage of resazurin reduction relative to the control (Ctrl); (**B**) Anti-inflammatory activity was measured as inhibition of NO production, quantified by the Griess assay. Nitrite concentration was determined from a sodium nitrite standard curve and the results are expressed as concentration (μM) of nitrite in a culture medium. Each value represents the mean ± SD from at least three independent experiments (** *p* < 0.01 compared to Ctrl; # *p* < 0.05, ### *p* < 0.001 compared to ethanol (EtOH, vehicle) plus lipopolysaccharide (LPS)).

The anti-inflammatory activity of the lipid extract of *Gracilaria* sp. was assessed based on its ability to inhibit nitric oxide (NO) production in RAW 264.7 macrophages stimulated with LPS. For a range of concentrations between 25 and 100 µg/mL, the lipid extract did not compromise the cellular viability of macrophages (Figure 4A). The extract showed a dose-dependent NO inhibition of 35% attained at the concentration of 100 µg/mL (Figure 4B). Therefore, the concentration exhibiting anti-inflammatory activity also presented a safety profile to macrophages (Figure 4A). Meanwhile, at lower concentrations (≤ 50 μg/mL), the extract had no significant inhibitory effect.

Previous works have reported that polar lipid may be beneficial for inflammatory diseases [11,20,55,56]. Accordingly, polar lipids isolated from red algae have demonstrated strong anti-inflammatory activity, even higher when compared with pure 20:5(*n*-3) FA isolated from the same extracts [43], suggesting that the polar lipid itself may contribute to the anti-inflammatory activity. In the cases of *Chondrus crispus* and *Palmaria palmata* (Linnaeus) Weber & Mohr, 1805, the polar lipids such as glycolipids and phospholipids showed NO inhibitory activity through downregulation of inducible nitric oxide synthase (iNOS) [20,21,57]. Moreover, extracts rich in glycolipids bearing high proportions of PUFA, isolated from the red seaweeds *Palmaria palmata*, *Porphyra dioica* J. Brodie & L. M. Irvine, 1997, and *Chondrus crispus*, downregulated LPS-induced pro-inflammatory responses in human macrophages through the inhibition of IL-6 and IL-8 production, thus inferring their potential anti-inflammatory activity [15]. Therefore, as for other red seaweeds, the lipid extract from *Gracilaria* sp. proved to have effective anti-inflammatory activity. 

Lipid extract from *Gracilaria* sp. showed antiproliferative and anti-inflammatory activity. However, it was not possible to determine exactly which lipid components are responsible for these bioactivities. Even in the literature, the majority of studies have also addressed biological activities of lipid extracts rather than pure lipid molecules, which hampers the determination of a relationship between structure and bioactivity. This is due to the fact that the isolation of a pure lipid molecule is very difficult and even pure lipid standards are not available for several lipid classes. Some authors have put some effort into this issue, scarcely addressed for the lipid extracts from seaweed, and isolated enriched extracts in some classes of lipids to further test their bioactivity. Ohta et al. [17] reported that SQDG (20:5/16:0), isolated from red seaweed *Gigartina tenella*, was a potent inhibitor of eukaryotic DNA polymerases [17]. Tsai et al. also reported that enriched extract with SQDG isolated from red seaweeds, with high levels of PUFAs such as 20:4(*n*-6) FA and 20:5(*n*-3) FA, inhibited the growth of human hepatocellular carcinoma cell line (HepG2), rather than enriched extracts with MGDG or DGDG [16]. This research group has also showed that the sulfolipids isolated from seaweed exhibited higher inhibitory effect than sulfolipids isolated from spinach, previously reported as inhibitors of DNA polymerases and of the proliferation of human cervix carcinoma (HeLa) [22]. The aforementioned SQDG-enriched extracts displayed strong inhibitory effects and contained SQDG (20:5/16:0) [17] or contained SQDG assembling PUFAs [16], which are also found in the extract of *Gracilaria* sp. analyzed within this work. Thus, SQDG (18:2/16:0), SQDG (18:4/16:0), SQDG (20:4/16:0), and SQDG (20:5/16:0), identified in the extract of *Gracilaria* sp., can contribute to the observed antiproliferative effects.

In what concern anti-inflammatory activities, Banskota et al. reported that the extracts rich in MGDG and DGDG isolated from red seaweed *Chondrus crispus* inhibited NO production through downregulation of iNOS [21]. The enriched extract contained MGDG (20:5/20:5), MGDG (20:5/20:4), MGDG (18:4/16:0), MGDG (20:4/16:0), and MGDG (20:5/16:0) and the respective DGDG analogues. Interestingly, the majority of these molecular species were also found in the extract of *Gracilaria* sp. analyzed in the present work. Moreover, the same group of researchers isolated MGDG, DGDG, SQDG, PC, and PG molecular species from the lipid extract of *Palmaria palmata* and all the polar lipids showed NO inhibitory activity [20]. The isolated polar lipids identified were MGDG (20:5/20:5), MGDG (20:5/16:0), DGDG (20:5/20:5), DGDG (20:5/14:0), DGDG (20:5/16:0), SQDG (20:5/14:0), PG (20:5/16:0), PG (20:5/16:1) and PC (20:5/20:5). All the molecular species contained 20:5(*n*-3) FA, and showed higher activity than the free FA 20:5(*n*-3), suggesting that the entire polar lipid structure (e.g., sulfolipid, phospholipid, or galactolipids) is essential for the extension of NO inhibition. Aside from the PC, the reported glycolipids and PG were also found in the lipidome of *Gracilaria* sp. Thus, the presence of these glycolipids and PGs in the lipid extract of *Gracilaria* sp. can contribute to the observed anti-inflammatory properties. 

The presence of several polar lipids with recognized bioactive polar lipids in *Gracilaria* sp. can be related to the bioactivity observed in this work. However, more studies are needed to understand the structural/bioactivity relation of seaweed polar lipids, which deserve to be explored.

## 3. Experimental Section

### 3.1. Biomass

Dried samples (25 °C, up to 12% moisture content) of *Gracilaria* sp. (*G. vermiculophylla* or *G. gracilis*, pending confirmation by DNA barcode analysis) (harvested in August 2014) were provided by ALGAplus Ltd. (production site located at Ria de Aveiro, mainland Portugal, 40°36′43″ N, 8°40′43″ W). The biomass is continuously produced by clonal propagation (asexual reproduction strategy) and thus has lower variability than would be expected from wild harvested biomass.

### 3.2. Reagents

HPLC grade chloroform and methanol were purchased from Fisher Scientific Ltd. (Loughborough, UK). All other reagents were purchased from major commercial sources. Milli-Q water (Synergy, Millipore Corporation, Billerica, MA, USA), RPMI 1640 media from PAA (Pasching, Austria), Phenol-red-free RPMI 1640 medium, penicillin–streptomycin, TrypLE express, fetal bovine serum (FBS), and Presto Blue from Gibco Technologies (Invitrogen Life Sciences, Paisley, UK) were used.

### 3.3. Lipid Extraction Procedure

A mixture of chloroform/methanol (1:2, *v*/*v*) was added to 250 mg of dry weight seaweed. The mixture was transferred to a glass tube with a Teflon-lined screw cap and, after the addition of 3.75 mL of solvent mixture, it was homogenized by vortexing for 2 min and then incubated in ice on an orbital shaker for 2 h 30 min. The mixture was centrifuged at 2000 rpm for 10 min and the organic phase collected. The biomass residue was re-extracted twice with 1.5 mL of solvent mixture and 2.3 mL of water was added to the total collected organic phase to induce phase separation. Following this procedure, samples were centrifuged for 10 min at 1500 rpm, and the organic (lower) phase was collected in a new tube. Three biological replicates were performed, with extractions and analyses taking place on different days. Lipid extracts were dried under a stream of nitrogen gas and the lipid content was estimated as (%) of dry weight. Lipid extracts were stored at −20 °C prior to analysis by LC–MS.

### 3.4. Quantification of Glycolipids and Phospholipids

Glycolipid quantification was achieved by calculating the hexose content (% glucose) through the orcinol colorimetric method (CyberLipids, [58]). The amount of sugar was read from a calibration curve prepared by performing the reaction on known amounts of glucose (up to 40 μg, from an aqueous solution containing 5 mg/mL of sugar). Phospholipids were quantified by a molybdovanadate method for the simultaneous assay of orthophosphate and some organic phosphates, as described by Bartlett and Lewis, and routinely performed in the authors’ laboratory [26,59,60]. Absorbance of standards and samples was measured on a microplate UV-Vis spectrophotometer (Multiskan GO, Thermo Scientific, Hudson, NH, USA).

### 3.5. Fractionation of Lipid Extract

Isolation of polar lipids from pigments was performed using a modification of Pacetti’s method [61]. A sample of lipid extract (1 mg) was dissolved in 300 μL of chloroform and transferred to a Supelclean™ LC–Si SPE Tube (bed wt. 500 mg, volume 3 mL cartridges; SUPELCO, Sigma–Aldrich, St. Louis, MO, USA), followed by sequential elution with 4 mL of chloroform, 3 mL of ether diethyl ether:acetic acid (98:2), 5 mL of acetone:methanol (9:1 *v*/*v*), and 4 mL of methanol. Fractions 1 and 2, corresponding to neutral lipids and pigments, were discarded. Fractions 3 and 4, rich in glycolipids and in phospholipids plus betaines, respectively, were recovered, separated, dried under nitrogen, and stored at −20 °C prior to analysis by ESI–MS.

### 3.6. Hydrophilic Interaction Liquid Chromatography–Electrospray Ionization–Mass Spectrometry (HILIC–ESI–MS)

Lipid extracts were analyzed by hydrophilic interaction liquid chromatography (HILIC) on a Waters Alliance 2690 HPLC system (Waters Corp., Milford, MA, USA) coupled to a Finnigan LXQ electrospray linear ion trap mass spectrometer (Thermo Fisher, San Jose, CA, USA). Mobile phase A consisted of 25% water, 50% acetonitrile, and 25% methanol, with 1 mM ammonium acetate, and mobile phase B consisted of 60% acetonitrile and 40% methanol with 1 mM ammonium acetate. Lipid extracts (12.5 μg) were diluted in mobile phase B (100 μL) and 10 μL of the reaction mixture were introduced into an Ascentis Si HPLC Pore column (15 cm × 1.0 mm, 3 μm; Sigma–Aldrich, St. Louis, MO, USA). The solvent gradient, flow rate through column and conditions used for acquisition of full scan LC–MS spectra and LC–MS/MS spectra in both positive and negative ion modes were the same as previously described [25,26]. The identification of molecular species of polar lipids was based on the assignment of the molecular ions observed in LC–MS spectra. Only ions observed in the LC–MS spectra with a relative abundance >2% were considered for identification. All analyses were performed in analytical triplicate.

### 3.7. Electrospray–Mass Spectrometry (ESI–MS) Conditions

Fractions 3 and 4 recovered from lipid extract were analyzed by ESI–MS on a Q-Tof 2 quadrupole time of flight mass spectrometer (Micromass, Manchester, UK) operating in positive mode. Each sample, diluted in 195 µL of methanol, was introduced through direct infusion with the following electrospray conditions: flow rate of 10 mL/min, voltage applied to the needle at 3 kV, a cone voltage at 30 V, source temperature of 80 °C, and solvation temperature of 150 °C [62]. The resolution was set to about 9000 FWHM (full width at half maximum). Tandem mass spectra (MS/MS) were acquired by collision induced dissociation (CID), using argon as the collision gas (pressure measured as the setting in the collision cell 3.0 × 10^5^ Torr). The collision energy was between 30 and 60 eV. Both MS and MS/MS spectra were recorded for 1 min. Data acquisition was carried out with a MassLynx 4.0 data system. 

### 3.8. Fatty Acid Analysis by Gas Chromatography-Mass Spectrometry (GC–MS)

Fatty acid methyl esters (FAMEs) were prepared from lipid extracts using a methanolic solution of potassium hydroxide (2.0 M) according to the methodology previously described [26]. Volumes of 2.0 μL of the hexane solution containing FAMEs were analyzed by gas chromatography-mass spectrometry (GC–MS) on an Agilent Technologies 6890 N Network (Santa Clara, CA, USA) equipped with a DB-FFAP column with the following specifications: 60 m long, 0.25 mm internal diameter, and 0.25 μm film thickness (J & W Scientific, Folsom, CA, USA). The GC equipment was connected to an Agilent 5973 Network Mass Selective Detector operating with an electron impact mode at 70 eV and scanning the range *m/z* 40–500 in a 1 s cycle in a full scan mode acquisition. The oven temperature was programmed from an initial temperature of 80 °C, a linear increase to 155 °C at 15 °C/min, followed by linear increase at 8 °C/min to 210 °C, then at 30 °C/min to 250 °C, standing at 250 °C for 18 min. The injector and detector temperatures were 220 and 280 °C, respectively. Helium was used as the carrier gas at a flow rate of 0.5 mL/min. The identification of each FA was performed by mass spectrum comparison with those in the Wiley 275 library and confirmed by its interpretation and comparison with the literature. The relative amounts of FAs were calculated by the percent area method with proper normalization, considering the sum of all areas of the identified FAs.

### 3.9. Cell Viability Assay on T-47D and 5637 Tumor Cell Lines

The antiproliferative activity of lipid extracts was examined by the effect of *Gracilaria* sp. lipid extracts on the T-47D human breast cancer and urinary bladder cancer cell lines’ metabolism using the Prestoblue colorimetric assay (Invitrogen Life Sciences, Paisley, UK). Tumor cells were cultivated in Dulbecco’s Modified Eagle Medium (DMEM-F12, Invitrogen Life Technologies, Paisley, UK) with 10% fetal bovine serum (FBS; Gold, PAA) and 5 mg/L 1% penicillin/steptomicin (Invitrogen) in a humidified incubator at 37 °C under an atmosphere of 5% CO_2_. Cell were plated on 96-well plates and allowed to attach for 24 h, 100 µL of cell suspension (1–2 × 10^4^ cell/mL in complete medium) were used. Following this step, 200 µL of the treatment solution in a range of 25–100 μg/mL were applied to the culture. The lipid extract was dissolved in DMSO and diluted to a final concentration of 0.1% DMSO in a phenol-red free RPMI 1640 medium supplemented with 2% charcoal treated FBS (DCC), 1% glutamate, and 1% PEST. The same concentration of DMSO was used in untreated controls [63]. The treatment medium was changed 48 h later, and was removed from each cell after 48 h for viability assay using PrestoBlue Absorbance measured at 570 nm and 600 nm at 1, 2, 3, 4, and 5 h on a plate reader, which gave a linear absorbance range. Experiments were carried out in quadruplicate and three independent experiments were carried out for each cell line.

### 3.10. Anti-Inflammatory Activity on Nitrite Production in RAW 264.7 Cells

Test solutions of *Gracilaria* sp. lipid extracts (25 mg/mL) were prepared in ethanol and stored at –20 °C until used. Serial dilutions of tested solutions with culture medium were prepared and sterilized by filtration immediately before in vitro assays. Ethanol concentrations ranged from 0.1% to 0.8% (*v*/*v*).

RAW 264.7, a mouse leukemic monocyte macrophage cell line from American Type Culture Collection (ATCC TIB-71), was supplied by Otília Vieira (Centro de Neurociências e Biologia Celular, Universidade de Coimbra, Coimbra, Portugal) and cultured in Dulbecco’s Modified Eagle Medium (Invitrogen Life Technologies, Paisley, UK) supplemented with 10% non-inactivated fetal bovine serum, 100 U/mL penicillin, and 100 μg/mL streptomycin at 37 °C in a humidified atmosphere of 95% air and 5% CO_2_. During the experiments, cells were monitored through microscope observation to detect any morphological change. Assessment of metabolically active cells was performed using a resazurin bioassay [64]. Briefly, cell duplicates were plated at a density of 0.1 × 10^6^/well, in a 96-well plate and allowed to stabilize overnight. Following this period, cells were either maintained in a culture medium (control) or pre-incubated with various concentrations of *Gracilaria* sp. lipid extracts or its vehicle for 1 h, and later activated with 50 ng/mL LPS for 24 h. After the treatments, resazurin solution (50 μM in culture medium) was added to each well and incubated at 37 °C for 1 h, in a humidified atmosphere of 95% air and 5% CO_2_. As viable cells are able to reduce resazurin (a non-fluorescent blue dye) into resorufin (pink and fluorescent), their number correlates with the magnitude of dye reduction. Quantification of resofurin was performed on a Biotek Synergy HT (BioTek Instruments, Winooski, VT, USA) plate reader at 570 nm, with a reference wavelength of 620 nm. The production of nitric oxide was measured by the accumulation of nitrite in the culture supernatants, using a colorimetric reaction with the Griess reagent [65]. Briefly, 170 μL of culture supernatants were diluted with equal volumes of the Griess reagent [0.1% (*w*/*v*) *N*-(1-naphthyl)-ethylenediamine dihydrochloride and 1% (*w*/*v*) sulphanilamide containing 5% (*w*/*v*) H_3_PO_4_] and maintained for 30 min in the dark. The absorbance at 550 nm was measured on a Biotek Synergy HT plate reader. Culture medium was used as a blank and nitrite concentration (μM) was determined from a regression analysis using serial dilutions of sodium nitrite as standard. Experiments were carried out at least three times.

### 3.11. Statistical Analysis

Antiproliferative and anti-inflammatory bioassays were measured in quadruplicate and in three different and independent experiments. Results were expressed as mean ± SD. One-way analysis of variance (ANOVA) followed by Dunnett’s multiple comparison tests was used to compare the treatment group to a single control group, after checking for assumptions. Statistical differences were calculated and represented with the following symbols of significance level ** *p* < 0.01, *** *p* < 0.001, # *p* < 0.05, ### *p* < 0.001. Statistical analysis was performed using GraphPad Prism 5.0 for Windows (GraphPad Software, San Diego, CA, USA).

## 4. Conclusions

The comprehensive elucidation of the *Gracilaria* sp. lipidome has been successfully accomplished for the first time. Liquid chromatography–mass spectrometry–based approach afforded the identification of 147 molecular species of polar lipids, distributed between the glycolipids, phospholipids, and betaine lipids classes. It was possible to identify novel sulfolipids (SQMG) and betaine lipids, among which DGTS were identified for the first time on the genus *Gracilaria* and MGTS within the Rhodophyta. Lipid extracts (~80% polar lipids) from *Gracilaria* sp. cultivated on land-based IMTA were screened for bioactivity and collectively shown to be a natural source of bioactive lipids with antiproliferative and anti-inflammatory activities. The presence of these bioactive polar lipids in *Gracilaria* sp. promotes its consumption as a functional food for the prevention of various diseases. Seaweeds’ land-based culture using IMTA is a sustainable solution towards the production of large volumes of biomass displaying replicable bioactive properties. The higher degree of production conditions control enabled by land-based IMTA, versus open-water large-scale culture, allows for the production of higher value products with better positioning in value-chains supplying high-end markets.

## Figures and Tables

**Table 1:** Composition of lipid extract of *Gracilaria* sp. (mean and SD of triplicate).

Composition	Mean	SD
Lipids (mg/kg biomass)	3000	600
Glycolipids (mg/kg biomass)	1980	148
Phospholipids (mg/kg biomass)	165	52.7
Betaines and others ^1^	855	-

^1^ Betaines and others were determined by the difference of lipid content and the sum of content of glycolipids and phospholipids.

**Table 2:** Identification of MGDG and DGDG molecular species observed by HILIC–ESI–MS, as [M + NH_4_]^+^ ions and SQDG and SQMG molecular species observed as [M ∓ H]^-^ ions ^2^.

**[M + NH_4_]^+^**	**Lipid Species**	**Fatty Acyl Chains**
***m/z***	**(C:N)**	
**Monogalactosyl diacylglyceride (MGDG)**		
746.3	MGDG (32:1)	16:1/16:0 and 14:0/18:1
748.3	MGDG (32:0)	16:0/16:0 and 14:0/18:0
**774.3**	**MGDG (34:1)**	18:1/16:0
776.3	MGDG (34:0)	18:0/16:0
794.3	MGDG (36:5)	20:5/16:0
**796.3**	**MGDG (36:4)**	20:4/16:0 and 18:2/18:2
**Digalactosyl diacylglyceride (DGDG)**		
908.3	DGDG (32:1)	16:1/16:0 and 14:0/18:1
910.3	DGDG (32:0)	16:0/16:0 and 14:0/18:0
934.3	DGDG (34:2)	18:2/16:0 and 18:1/16:1
**936.3**	**DGDG (34:1)**	18:1/16:0
956.3	DGDG (36:5)	20:5/16:0
**958.3**	**DGDG (36:4)**	20:4/16:0 and 18:2/18:2
**[M − H]^−^**	**Lipid Species**	**Fatty Acyl Chains**
**Sulfoquinovosyl diacylglyceride (SQDG)**		
763.6	SQDG (30:1)	14:0/16:1
**765.6**	**SQDG (30:0)**	14:0/16:0
791.6	SQDG (32:1)	16:1/16:0 and 14:0/18:2
**793.6**	**SQDG (32:0)**	16:0/16:0 and 14:0/18:0
813.6	SQDG (34:4)	18:4/16:0
817.6	SQDG (34:2)	18:2/16:0
819.6	SQDG (34:1)	18:1/16:0
839.6	SQDG (36:5)	20:5/16:0
**841.6**	**SQDG (36:4)**	20:4/16:0
857.6	SQDG (36:4-OH)	20:4-OH/16:0
**Sulfoquinovosyl monoacylglyceride (SQMG)**		
527.4	SQMG (14:0)	
553.4	SQMG (16:1)	
555.4	SQMG (16:0)	

^2^ The assignment of the fatty acyl composition of molecular species was made according to the interpretation of the corresponding MS/MS spectra. Bold *m/z* values correspond to the most abundant species detected in the LC–MS spectrum; C means the number of carbon atoms; N represents double bonds in the fatty acyl chains; MGDG: monogalactosyl diacylglyceride; DGDG: digalactosyl diacylglyceride; SQMG: sulfoquinovosyl monoacylglyceride; SQDG: sulfoquinovosyl diacylglyceride; and HILIC–ESI–MS: hydrophilic interaction liquid chromatography–electrospray ionization–mass spectrometry.

**Table 3:** Identification of phospholipid molecular species observed by HILIC–ESI–MS, as [M + H]^+^ ions for PC, LPC, and PE and as [M − H]^−^ ions for PG, LPG, PI, PA, and IPC ^2^.

**[M + H]^+^**	**Lipid Species**	**Fatty Acyls Chain**
***m/z***	**(C:N)**	
**Phosphatidylcholine (PC)**		
732.6	PC (32:1)	16:0/16:1 and 14:0/18:1
734.6	PC (32:0)	16:0/16:0 and 14:0/18:0
754.6	PC (34:4)	14:0/20:4 and 16:2/18:2
756.6	PC (34:3)	16:0/18:3 and 14:0/20:3
758.6	PC (34:2)	16:0/18:2 and 16:2/18:1
**760.6**	**PC (34:1)**	16:0/18:1
762.6	PC (34:0)	16:0/18:0
780.6	PC (36:5)	16:0/20:5 and 18:2/18:3
**782.6**	**PC (36:4)**	16:0/20:4 and 18:2/18:2
784.6	PC (36:3)	16:0/20:3 and 18:1/18:2
786.6	PC (36:2)	18:0/18:2 and 18:1/18:1
788.6	PC (36:1)	18:0/18:1
798.5	PC (37:3)	16:0/21:3 and 18:1/19:2
804.5	PC (38:7)	18:3/20:4 and 18:2/20:5
806.5	PC (38:6)	18:2/20:4 and 18:1/20:5
808.5	PC (38:5)	18:1/20:4 and 18:2/20:3
810.5	PC (38:4)	18:1/20:3 and 16:0/22:4
812.5	PC (38:3)	18:0/20:3 and 18:1/20:2
814.5	PC (38:2)	16:0/22:2 and 18:1/20:1
818.5	PC (38:0)	18:0/20:0 and 16:0/22:0
840.4	PC (40:3)	18:1/22:2
844.4	PC (40:1)	18:1/22:0
**Lyso-phosphatidylcholine (LPC)**		
494.4	LPC (16:1)	
496.4	LPC (16:0)	
518.4	LPC (18:3)	
520.4	LPC (18:2)	
522.4	LPC (18:1)	
524.4	LPC (18:0)	
542.4	LPC (20:5)	
**544.4**	**LPC (20:4)**	
**Phosphatidyletanolamine (PE)**		
**716.4**	**PE (34:2)**	16:1/18:1 and 16:0/18:2
718.3	PE (34:1)	16:1/18:0 and 16:0/18:1
740.4	PE (34:0)	16:0/18:0
742.4	PE (36:3)	18:1/18:2
744.4	PE (36:2)	18:1/18:1
746.3	PE (36:1)	18:0/18:1
**[M − H]^−^**	**Lipid Species**	**Fatty Acyl Chains**
**Phosphatidylglycerol (PG)**		
717.4	PG (32:2)	16:1/16:1 and 16:0/16:2
719.4	PG (32:1)	16:0/16:1
721.4	PG (32:0)	16:0/16:0
741.4	PG (34:4)	16:0/18:4
743.5	PG (34:3)	16:0/18:3
745.5	PG (34:2)	16:1/18:1
747.5	PG (34:1)	16:0/18:1 and 16:1/18:0
767.5	PG (36:5)	16:0/20:5
**769.4**	**PG (36:4)**	16:0/20:4 and 18:2/18:2
773.5	PG (36:2)	18:1/18:1
**Lyso-phosphatidylglycerol (LPG)**		
481.3	LPG (16:1)	
**483.3**	**LPG (16:0)**	
509.3	LPG (18:1)	
531.3	LPG (20:4)	
**Phosphatidylinositol (PI)**		
833.5	PI (34:2)	16:1/18:1
835.5	PI (34:1)	16:0/18:1
**Phosphatidic acid (PA)**		
693.4	PA (36:5)	16:0/20:5
695.4	PA (36:4)	16:0/20:4
717.4	PA (38:7)	18:3/20:4
719.4	PA (38:6)	18:2/20:4
721.4	PA (38:5)	18:1/20:4
741.3	PA (40:9)	20:4/20:5
**743.3**	**PA (40:8)**	20:4/20:4
745.3	PA (40:7)	20:3/20:4
**Inositolphosphoceramide (IPC)**		
**810.5**	**IPC (*t*35:0)**	*t*18:0/17:0
908.6	IPC (*d*42:0)	*d*18:0/24:0
**920.6**	**IPC (*t*42:2)**	*t*18:1/24:1
922.6	IPC (*t*42:1)	*t*18:0/24:1
924.6	IPC (*t*42:0)	*t*18:0/24:0

^2^ The assignment of the fatty acyl composition of molecular species was made according to the interpretation of the corresponding MS/MS spectra. Bold *m/z* values correspond to the most abundant species detected in the LC–MS spectrum; C means the number of carbon atoms; N represents double bonds in the fatty acyl chains; PC: phosphatidylcholine; LPC: lyso-PC; PG: phosphatidylglycerol; LPG: lyso-PG; PI: phosphatidylinositol; PA: phosphatic acid; PE: phosphatidylethanolamine; and IPC: inositolphosphoceramide.

**Table 4:** Identification of DGTS and MGTS molecular species observed by HILIC–ESI–MS as [M + H]^+^ ions ^2^.

**[M + H]^+^**	**Lipid Species**	**Fatty Acyls Chain**
***m/z***	**(C:N)**	
**Diacylglyceryl trimethyl homoserine (DGTS)**		
656.7	DGTS (28:0)	14:0/14:0
682.7	DGTS (30:1)	14:0/16:1
684.8	DGTS (30:0)	14:0/16:0
708.7	DGTS (32:2)	16:1/16:1 and 14:0/18:2
**710.7**	**DGTS (32:1)**	16:0/16:1 and 14:0/18:1
712.7	DGTS (32:0)	16:0/16:0 and 14:0/18:0
732.7	DGTS (34:4)	16:2/18:2 and 14:0/20:4
734.7	DGTS (34:3)	16:1/18:2
736.7	DGTS (34:2)	16:0/18:2 and 16:1/18:1
738.7	DGTS (34:1)	16:0/18:1 and 16:1/18:0
740.7	DGTS (34:0)	16:0/18:0 and 14:0/20:0
760.6	DGTS (36:4)	16:0/20:4
**764.8**	**DGTS (36:2)**	18:1/18:1
766.8	DGTS (36:1)	18:0/18:1
**Monoacylglyceryl trimethyl homoserine (MGTS)**		
446.5	MGTS (14:0)	
472.5	MGTS (16:1)	
474.5	MGTS (16:0)	
498.6	MGTS (18:2)	
500.6	MGTS (18:1)	

^2^ The assignment of the fatty acyl composition of molecular species was made according to the interpretation of the corresponding MS/MS spectra. Bold *m/z* values correspond to the most abundant species detected in the LC–MS spectrum; C means the number of carbon atoms; N represents double bonds in the fatty acyl chains; MGTS: monoacylglyceryl-*N*,*N*,*N*-trimethyl homoserine; and DGTS: diacylglyceryl-*N*,*N*,*N*-trimethyl homoserine.

## Supplementary Materials

LC–MS spectra information are available online at www.mdpi.com/1660-3397/15/3/62/s1.
