# Supplementary material for: Valorization of Lipids from Gracilaria sp. through Lipidomics and Decoding of Antiproliferative and Anti-Inflammatory Activity
Source: Mar Drugs. 2017 Mar 2;15(3):62. doi: 10.3390/md15030062 (PMC5367019; doi:10.3390/md15030062)
Supplement: Supplementary file 1 [file marinedrugs-15-00062-s001.pdf]

Supplementary information: Valorization of lipids from *Gracilaria* sp. trough lipidomics approach and decoding of antiproliferative and anti-inflammatory activities

Elisabete da Costa, Tânia Melo, Ana S. P. Moreira, Carina Bernardo, Luisa Helguero, Isabel Ferreira, Maria Teresa Cruz, Andreia M. Rego, Pedro Domingues, Ricardo Calado, Maria H. Abreu and Maria Rosário Domingues

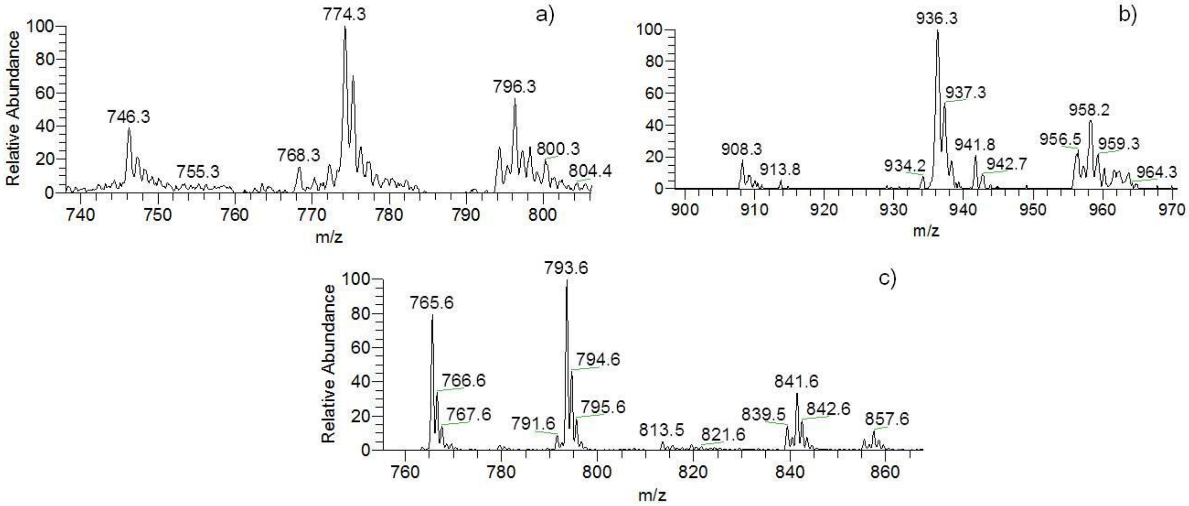

Figure S1. LC–MS spectra of the classes (a) monogalactosyl diacylglyceride (MGDG) and (b) digalactosyl diacylglyceride (DGDG) observed as $[M + NH_4]^+$, and (c) sulfoquinovosyl diacylglyceride (SQDG) observed as $[M - H]^-$.

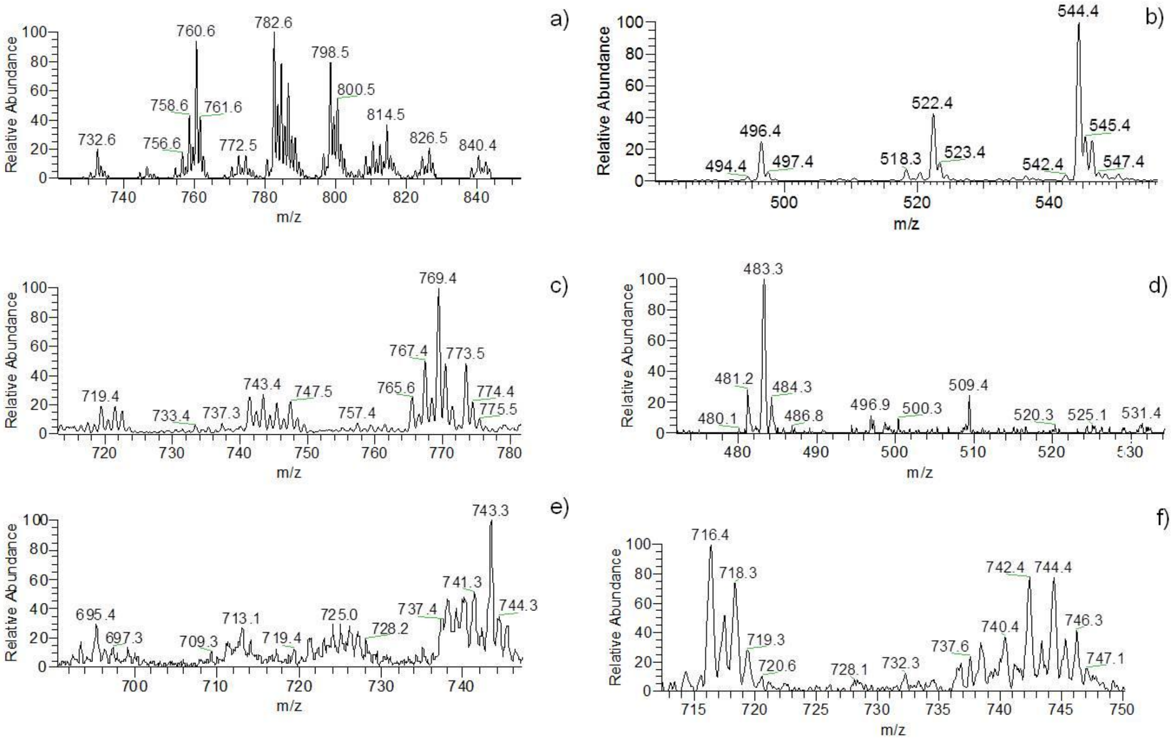

Figure S2. LC–MS spectra of the classes (a) phosphatidylcholine (PC) and (b) lyso phosphatidylcholine (LPC) observed as $[M + H]^+$ ions; (c) phosphatydilglycerol (PG) and (d) lyso phosphatidylglycerol (LPG) observed as $[M - H]^-$ ions; (e) phosphatidic acid (PA) observed as $[M - H]^-$ ions and f) phosphatidilethanolamine (PE) observed as $[M + H]^+$ ions.

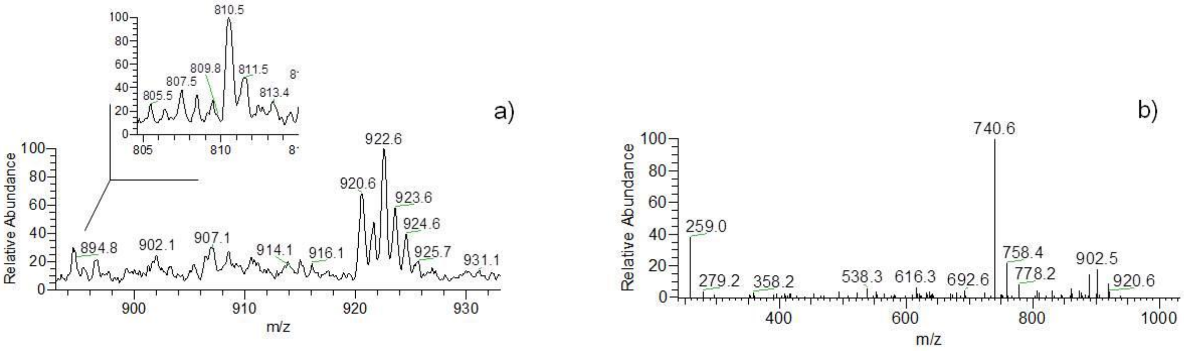

Figure S3. (a) LC–MS spectrum of inositolphosphoceramide (IPC), negative mode, observed as $[M - H]^-$. ions; (b) LC–MS/MS spectrum of $[M - H]^-$ ions of IPC at *m*/*z* 920.6 (IPC (*t*18:1/24:1), showing the typical fragmentation pathways of IPCs such as the loss of 162 Da and 180 Da due to the loss of the inositol residue and inositol moiety, respectively; product ion at *m*/*z* 538.3 resulting from the loss of fatty acyl chain and the product ion at *m*/*z* 259 corresponding to an inositol-monophosphate anion.

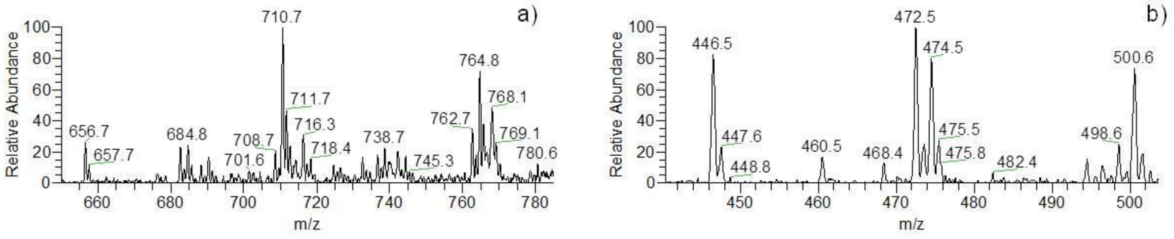

Figure S4. LC–MS spectra of (a) diacylglyceryl-*N*,*N*,*N*-trimethylhomoserine specie (DGTS) and (b) monoacylglyceryl-*N*,*N*,*N*-trimethylhomoserine (MGTS), positive modes, observed as $[M + H]^+$.
